# High Levels of Variation Within Gene Sequences of *Olea europaea* L.

**DOI:** 10.3389/fpls.2018.01932

**Published:** 2019-01-08

**Authors:** Nicolò G. M. Cultrera, Vania Sarri, Livia Lucentini, Marilena Ceccarelli, Fiammetta Alagna, Roberto Mariotti, Soraya Mousavi, Consolacion Guerrero Ruiz, Luciana Baldoni

**Affiliations:** ^1^Institute of Biosciences and Bioresources, National Research Council, Perugia, Italy; ^2^Department of Chemistry, Biology and Biotechnology, University of Perugia, Perugia, Italy; ^3^ENEA Italian National Agency for New Technologies Energy and Sustainable Economic Development, Trisaia Research Center, Rotondella, Italy

**Keywords:** sequencing, *ACP*, *LUS*, *SUT*, SNP, genotyping

## Abstract

Gene sequence variation in cultivated olive (*Olea europaea* L. subsp. *europaea* var. *europaea*), the most important oil tree crop of the Mediterranean basin, has been poorly evaluated up to now. A deep sequence analysis of fragments of four genes, *OeACP1*, *OeACP2, OeLUS* and *OeSUT1*, in 90 cultivars, revealed a wide range of polymorphisms along all recognized allele forms and unexpected allele frequencies and genotype combinations. High linkage values among most polymorphisms were recorded within each gene fragment. The great sequence variability corresponded to a low number of alleles and, surprisingly, to a small fraction of genotype combinations. The distribution, frequency, and combination of the different alleles at each locus is possibly due to natural and human pressures, such as selection, ancestrality, or fitness. Phylogenetic analyses of allele sequences showed distant and complex patterns of relationships among cultivated olives, intermixed with other related forms, highlighting an evolutionary connection between olive cultivars and the *O. europaea* subspecies *cuspidata* and *cerasiformis*. This study demonstrates how a detailed and complete sequence analysis of a few gene portions and a thorough genotyping on a representative set of cultivars can clarify important issues related to sequence polymorphisms, reconstructing the phylogeny of alleles, as well as the genotype combinations. The identification of regions representing blocks of recombination could reveal polymorphisms that represent putatively functional markers. Indeed, specific mutations found on the analyzed *OeACP1* and *OeACP2* fragments seem to be correlated to the fruit weight.

## Introduction

Cultivated olive (*Olea europaea* L. subsp. *europaea* var. *europaea*) is the most important oil tree crop of the Mediterranean basin. The species is characterized by an unusually rich germplasm, represented by a high number of traditional varieties, vegetatively propagated over 1000s of years (Baldoni et al., 2006), showing a patchy pattern of geographical distribution (Besnard et al., 2013; Diez et al., 2015; Mousavi et al., 2017). The origin and distribution of this genetic variation in the Mediterranean basin remain unclear. According to the most accredited hypotheses, the olive tree cultivation was introduced from the Near East, recognized as the first center of domestication during the early Neolithic period (Kaniewski et al., 2012; Zohary et al., 2012; Besnard et al., 2013, 2018; Mousavi et al., 2017). Multiple domestication events, probably, took place in different areas of the Mediterranean basin, with crosses among ancient varieties and wild plants (*O. europaea* subsp. *europaea* var. *sylvestris*, named as oleaster), followed by empirical local selection, probably associated to episodic varietal substitution, leading to the current varietal framework (Breton et al., 2009; Besnard et al., 2013, 2018; Hosseini-Mazinani et al., 2014; Diez et al., 2015).

Since this complex germplasm represents a valuable source of useful traits, a great effort is required to explore, manage, characterize and identify all genotypes. Molecular markers currently available for such purposes are still represented by some di-nucleotide microsatellites (Baldoni et al., 2009), small sets of poly-nucleotide EST-SSRs recently developed (de la Rosa et al., 2013a; Mariotti et al., 2016; Arbeiter et al., 2017) and lately by few nuclear SNPs (single nucleotide polymorphisms). These markers were identified on olive gene sequences and they have been used for cultivar genotyping (Reale et al., 2006; Consolandi et al., 2007; Hakim et al., 2009; Rossi et al., 2012; Kaya et al., 2013; Sabetta et al., 2013; Kalogianni et al., 2015; Uncu et al., 2015; Bazakos et al., 2016), or to reconstruct the phylogenetic relationships within the olive complex (Besnard et al., 2007; Besnard and El Bakkali, 2014; Biton et al., 2015).

Recently, next-generation sequencing technologies have been applied to discover polymorphisms at transcriptome and whole-genome level (Unver et al., 2017; Belaj et al., 2018). The efficiency of sequencing and SNP genotyping methodologies has rapidly increased, therefore, it is reasonable to expect that, due to their ubiquity in the genome, SNPs will soon become valuable markers for olive cultivar identification (Ben-Ari and Lavi, 2012; Cruz et al., 2016; Ipek et al., 2016; Marchese et al., 2016; Belaj et al., 2018). However, a high level of genome sequence information is required to identify and validate SNPs. For this reason, it is necessary to expand knowledge on the level and distribution of polymorphisms within gene sequences, mainly when genome data are still incomplete and referred to a few genotypes, as is the case of olive (Cruz et al., 2016; Unver et al., 2017). Valuable information on gene structure and allele arrangement will allow the discrimination among gene copies, as well as the identification of polymorphisms with a potential functional value (Swanson-Wagner et al., 2010; Lamesch et al., 2012; Luo et al., 2017; Mourad et al., 2018).

As already quoted, SNPs can be used to clarify the phylogenetic relationships among taxa (Andrello et al., 2017; Zhou et al., 2018), whereas gene phylogenies allowed to investigate the impact of selective pressure on single genes or members of gene families (Doebley et al., 2006; Fischer et al., 2011). A deep analysis of nuclear gene sequences could also reveal patterns of genome diversity useful for detecting selective sweeps or documenting the impact of genetic bottlenecks during domestication events (Wright et al., 2005; Fu, 2012; Liao et al., 2013; Nimmakayala et al., 2014; Huber et al., 2016; Marsden et al., 2016).

Taking this into account, an in-depth characterization of sequence portions of four genes in olive was undertaken in this work. Two acyl carrier protein (*ACP*) genes (Cultrera et al., 2014; De Marchis et al., 2016), a gene encoding for the sucrose transporter 1 (*SUT1*) (Conde et al., 2007) and another for lupeol synthase (*LUS*) (Alagna et al., 2015) were chosen on the basis of their proven functional role. Products of *ACPs* are essential cofactors involved in chloroplast fatty acid (FA) synthesis and desaturation, as well as in other metabolic pathways (Harwood, 1996; Suh et al., 1999; Byers and Gong, 2007; Li-Beisson et al., 2010; Tranbarger et al., 2011). The functional study of olive *ACP* loci showed that *OeACP2* was mainly expressed during fruit development, suggesting its involvement in FA synthesis for triacylglycerol accumulation (Cultrera et al., 2014), meanwhile the overexpression of *OeACP1* in tobacco plants resulted in a significant increase of oleic and linolenic acids (De Marchis et al., 2016). The *SUT1* gene codes for a protein representing a central machinery for mediating sucrose cross-membrane transport into cells and distributing sugars throughout the plant (Dusotoit-Coucaud et al., 2009). Different loci have been identified among woody plants (Decourteix et al., 2006; Fan et al., 2009; Afoufa-Bastien et al., 2010). In olive, only a partial complementary DNA clone of *SUT1* has been characterized (Conde et al., 2007; Alagna et al., 2012). The *LUS* gene, coding for an oxidosqualene cyclase (Shibuya et al., 1999; Sawai et al., 2006; Geisler et al., 2011), is involved in the production of a triterpene compound that strongly influences olive oil health quality (Saleem, 2009). Up to now, its sequence has been the most widely investigated in olive to search for polymorphisms useful for genotyping purposes (Reale et al., 2006; Hakim et al., 2009; Besnard and El Bakkali, 2014).

Polymorphic fragments of these genes were deeply analyzed within a large set of olive varieties, with the aims to: (i) understand the level of genomic variation within the chosen fragments; (ii) assign the alleles to each olive varieties; (iii) understand evolutionary and phylogenetic relationships among haplotypes, and (iv) verify potential correlations between detected polymorphisms and phenotypic traits.

## Materials and Methods

### Plant Material

A set of 90 olive varieties was included in the analysis (Table 1). DNA samples were derived from the olive DNA repository established at CNR – Institute of Biosciences and Bioresources (Perugia, Italy). Genetic identity of most varieties was previously ascertained through the use of well-established SSR markers (Sarri et al., 2006; Baldoni et al., 2009), whereas the others were directly genotyped for this work by means of the same set of markers (data not shown). Taking into account the clonal origin of olive cultivars, a single representative per cultivar was considered. To verify data repeatability, cultivars Oueslati and Zaity were analyzed twice. Samples of *O. europaea* subsp. *cuspidata* from Nepal and subsp. *cerasiformis* from the Canary Islands were used to clarify the ancestry and the phylogenetic relationships of the cultivar allele groups, whereas one sample of the related species *Olea paniculata*, belonging to the subgenus *Paniculatae* (Green, 2002), was used as an outgroup. Subspecies and *O. paniculata* samples also derived from the CNR-IBBR DNA repository.

**Table 1:** List of the olive cultivars investigated.

Cultivar	*OeACP1*		*OeACP2*		*OeLUS*		*OeSUT1*		Fruit purpose^∗^	Country of cultivation
Sigoise	1A	1A	4A	4A	C	D	S1	M2	a, b	Algeria
Istarka Belica	1A	1A	2D	2LA	C	D	M2	M2	a	Croatia
Oblica	1A	1A	2D	4A	C	C	M3	M3	a, b	Croatia
Toffahi	3C	3C	4A	4A	B	B	M7	L1	b	Egypt
Boutellan	1A	3D	2A	4A	C	C	M2	M2	a, b	France
Lucques	1A	1A	2D	4A	C	C	S4	M1	b	France
Oliviere	1A	3C	2LA	4A	C	C	S4	L1	a	France
Picholine	1A	3A	4A	4A	C	C	S4	M2	a, b	France
Verdale	1A	1A	2D	4A	B	C	M2	M2	a	France
Adramitini	1A	3E	2D	2LA	C	C	M2	M2	a	Greece
Amigdalolia	1A	3D	2D	4A	C	C	M2	M2	a, b	Greece
Kalamata	1A	3D	2D	4A	C	C	S3	M2	b	Greece
Kerkyras	1A	3B	4F	4L	E	F	S4	M2	a	Greece
Konservolia	1A	1A	2A	4A	C	D	M8	L1	b	Greece
Koroneiki	1A	3C	4D	4F	A	C	S4	M2	a	Greece
Mastoidis	1A	1A	2D	4F	D	G	M2	M2	a	Greece
Maureya	1A	1A	2LA	4A	A	C	S4	M2	b	Greece
Mirtolia	1A	1A	2A	4F	C	E	S4	M3	a	Greece
Merhavia	3D	3E	2A	4A	C	D	M2	M2	b	Israel
Ascolana Tenera	1A	3D	2LA	4A	C	C	S4	M2	b	Italy
Biancolilla	1A	3D	4F	4L	A	B	S3	M2	a	Italy
Borgiona	1A	3D	2D	4A	C	C	M2	M2	a	Italy
Bosana	1A	3C	2D	2LA	C	C	M2	M13	a	Italy
Caiazzana	1A	1A	2A	4C	D	E	S4	S4	a	Italy
Canino	1A	3C	4C	4F	C	C	S4	M1	a	Italy
Capolga	3C	3D	4F	4L	A	B	M1	L2	a	Italy
Cariasina	1A	3D	2D	2D	B	C	M2	M2	a, b	Italy
Carolea	1A	3C	2A	4A	C	C	M1	L2	a, b	Italy
Cassanese	1A	1A	2LA	4A	B	D	S4	M1	a, b	Italy
Cellina di Nardò	1A	1A	2C	2LA	C	D	S4	L1	a	Italy
Coratina	1A	3C	4C	4D	C	C	S4	M2	a	Italy
Dolce Agogia	1A	1A	2A	4C	E	F	S4	M2	a	Italy
Frantoio	1A	1A	2D	4C	C	D	S4	M2	a	Italy
Gargnà	1A	1A	2D	2D	C	D	S4	M1	a	Italy
Gentile di Chieti	1A	1A	2C	2D	C	C	M2	M2	a	Italy
Itrana	1A	1A	4F	4L	A	C	M1	L2	a, b	Italy
Leccino	1A	3C	2D	4A	C	C	S4	M2	a	Italy
Mignola Cartoceto	1A	3C	2C	2D	A	C	S4	M1	a	Italy
Moraiolo	1A	1A	4D	4F	A	C	S4	M2	a	Italy
Nocellara del Belice	1A	1A	2D	4A	A	B	M2	M2	b	Italy
Nostrale di Rigali	1A	1A	2D	4A	C	C	M2	M13	a	Italy
Nostrana di Brisighella	1A	1A	2C	4A	C	C	M3	L2	a	Italy
Olialonga	1A	3E	2C	2D	B	C	M2	M14	b	Italy
Orbetana	1A	1A	4A	4D	A	C	S4	M2	a	Italy
Ottobratica	1A	3C	2A	4A	C	D	S4	M3	a	Italy
Passalunara	1A	3D	2D	4A	C	C	M2	M2	a, b	Italy
Piantone di Mogliano	1A	3D	2D	4A	C	C	L2	M1	a	Italy
Pizz’e Carroga	1A	3E	4A	4A	B	C	M2	M13	a, b	Italy
Raia	1A	1A	2C	2D	C	D	S4	M2	a	Italy
Raio	1A	1A	2A	2A	C	D	M3	M2	a	Italy
Rosciola Colli Esini	1A	3B	2D	4H	A	C	S4	M2	a	Italy
Semidana	1A	3D	2D	4A	B	C	M2	M2	a	Italy
Sinopolese	1A	3C	2A	2A	C	C	S4	M3	a	Italy
Tonda Iblea	1A	1A	2LA	4A	C	C	M1	L2	b	Italy
Zaituna	3D	3E	2LA	4A	C	C	M2	M2	b	Italy
Picholine Marocaine	1A	1A	2LA	4A	C	D	M2	M13	a, b	Morocco
Galega	1A	3A	2LA	4A	C	C	S4	L1	a	Portugal
Arbequina	1A	3C	2D	2E	C	C	S2	M2	a	Spain
Blanqueta	1A	1A	2D	4A	C	C	S4	M2	a	Spain
Changlot Real	1A	3E	2A	2LB	B	D	S4	M3	a	Spain
Cornezuelo de Jaen	1A	3E	2LA	4A	B	D	M2	M2	a, b	Spain
Cornicabra	1A	1A	2D	4L	C	D	S4	M2	a	Spain
Empeltre	3B	3E	2A	4A	C	C	S4	M3	a	Spain
Farga	1A	3E	2C	2D	D	E	S4	L2	a	Spain
Gordal Sevillana	1A	3D	2D	4A	B	C	M2	L1	b	Spain
Hojiblanca	1A	3D	2D	2D	C	D	S3	M2	a, b	Spain
Lechin de Granada	1A	3E	2D	2LA	C	D	S4	M2	a	Spain
Lechin de Sevilla	1A	3C	4C	4C	B	C	S4	M2	a	Spain
Manzanilla Cacerena	1A	1A	2LB	4A	C	C	M2	L1	a, b	Spain
Manzanilla de Jaen	1A	3E	2LA	4A	C	D	S4	L1	b	Spain
Manzanilla de Sevilla	1A	1A	2LA	4A	C	C	S4	M1	b	Spain
Picual	1A	3D	2D	4A	C	C	M2	M9	a	Spain
Picudo	3D	3E	2LA	4A	C	D	S4	L1	a	Spain
Royal de Cazorla	1A	1A	2D	4A	C	D	S4	L1	a	Spain
Sevillenca	3D	3E	4F	4L	C	C	S4	M2	a	Spain
Verdial de Huevar	1A	1A	2D	4A	B	D	S4	L2	a	Spain
Villalonga	3D	3E	2LA	4A	C	D	S4	L1	a	Spain
Kaissy	3B	3E	2A	4A	C	B	M3	M3	a, b	Syria
Hrai Souni	1A	3C	4A	4C	B	C	S4	M2	a	Syria
Zaity	1A	1A	2D	4I	B	D	M2	M2	a	Syria
Chemlali	1A	1A	2A	4F	B	D	M1	L2	a	Tunisia
Meski	1A	1A	2LA	4A	B	C	M2	M2	b	Tunisia
Oueslati	1A	3C	2D	4C	A	C	S4	M2	a	Tunisia
Zalmati	1A	3C	4F	4L	A	C	S4	M2	b	Tunisia
Ayvalik	1A	3E	2D	2LA	C	C	M2	M2	a	Turkey
Elmacik	1A	3D	2D	2D	B	D	M9	L1	a	Turkey
Izmir Sofralik	1A	1A	2A	4A	C	D	M2	M2	b	Turkey
Memecik	1A	3D	4F	4L	A	C	S3	M2	a, b	Turkey
Uslu	3D	3E	2A	4A	B	B	M3	L2	b	Turkey
Yun Celebi	1A	3A	2A	4A	D	D	M6	L1	a	Turkey


*Alleles at the four loci and Country of cultivation of plant material are reported. For each cultivar, fruity purpose (a = oil, b = table) are given. ^∗^Information on fruit and purpose, was collected from www.oleadb.eu database and World Olive Cultivars Catalogue (Barranco et al., 2000).*

### PCR Amplification and Haplotype Characterization

The different gene portions were amplified from DNA of cv. Leccino, considering the availability of a good alignment of the genomic sequence of this cultivar (Barghini et al., 2014; Muleo et al., 2016).

The isolation of four *OeACP1* and *OeACP2* alleles was described in Cultrera et al. (2014), and their sequences were deposited in NCBI database (Accession Nos. KF303531–KF303532–KF303533–KF303534, respectively). The allele alignment for each gene allowed the detection of the polymorphic regions. A region was selected which includes different types of polymorphisms, including part of the first intron, the entire second exon and a large part of the second intron. Locus-specific primers were designed to amplify this partial gene sequences.

In order to obtain polymorphic *LUS* and *SUT1* gene fragments, primers were designed on their cDNA sequences (AB025343.1 and DQ087178.1 at GenBank, respectively).

Amplifications were initially performed on a subset of 20 cultivars. PCR amplifications were carried out in a reaction volume of 25 μl containing 25 ng of template DNA, 2.5 μl of 10× PCR buffer, 0.5 mM of each dNTP, 1 μM of each primer and 1.0 U of GoTaq (Promega). Amplifications were performed on a thermal cycler PCR System 9600 (Applied Biosystems, Foster City, CA, United States), using the following cycling conditions: initial denaturation at 95°C for 5 min, followed by 50 cycles of 95°C for 30 s, 59°C for 30 s and 72°C for 90 s, with a final elongation at 72°C for 10 min. Amplicons of different lengths were obtained. Considering that different fragment lengths prevent direct sequencing, in order to identify and distinguish the two alleles at each of the 20 diploid genotypes, amplicons of each locus were cloned, by using pGEM-T Easy Vector (Promega) and *E. coli* XL1 blue strain. DNA from 10 colonies for each genotype was amplified using the ExTaq (Takara) following the manufacturer instructions for the reaction and PCR amplification. Direct sequencing in both directions of the PCR products was performed on an ABI 3130 Genetic Analyzer (Applied Biosystems-Hitachi, United States) using the ABI Prism BigDye Terminator v.3.1 Ready Reaction Cycle Sequencing Kit (Applied Biosystems). To evaluate the presence of polymorphisms, sequences were aligned by BioEdit 7.1.7 (BioEdit^1^) and by Geneious 6.1.6 (Geneious^2^). Afterward, new allele-specific primers were designed and applied to amplify distinct alleles at each locus in all 90 cultivars (Supplementary Table S1). The PCR products were directly sequenced. For varieties carrying alleles of the same length but showing nucleotide polymorphisms, their amplicons were cloned again, in order to assign each of them to a distinct allele.

In order to verify whether the obtained alleles effectively belong to a single locus, a co-segregation analysis was performed by genotyping 94 individuals of a F1 intervarietal cross progeny (Leccino × Dolce Agogia), through length and SNP markers at each locus heterozygous in at least one parental variety (data not shown).

The same cloning strategy was also applied to obtain at least one allele for each sample of the *O. europaea* subspecies *cuspidata* and *cerasiformis* and the related species *O. paniculata*.

The obtained sequences were used as queries in a BLAST search on the genome of the cultivars Farga (^3^Cruz et al., 2016) and Leccino (^4^Muleo et al., 2012), and *O. europaea* subsp. *europaea* var. *sylvestris*^5^^,^^6^, in order to find the position of these genes on the chromosome.

### Data Analysis

At each locus, the number of SNPs, indels, SSRs and repeats was calculated. The frequency of polymorphisms was obtained by the ratio between number of polymorphisms and total fragment length. Linkage analysis among polymorphisms was evaluated through MapMaker/EXP Version 3.0b (Sharma and Kaur, 2014).

DnaSP was used to calculate the linkage disequilibrium (LD) among polymorphisms at each locus. The degree of LD was estimated by calculating r^2^ parameters and computing a non-linear regression line. The 180 fragments sequenced for each gene were analyzed, as well as the haplotypes inferred by the software. Due to the nature of the analyzed fragments, in this manuscript the terms ‘allele’ and ‘haplotype’ have been used as synonymous. The same software was used to detect the degree of intragenic recombination (IR) in real and inferred haplotypes at the four loci using genotypic data with the r^2^ parameter. The same software was used for haplotype reconstruction which was implemented by PHASE with 1,000 iterations, thinning intervals equal to 10 and 1,000 burn-in iterations, and either forcing or not the program to use the naive Gibbs sampler (GS options) (Stephens and Donnelly, 2003). The two-tailed Fisher’s exact test and the chi-square test were used to determine whether the associations between polymorphic sites were significant.

Median joining phylogenetic networks based on SNP and length markers for each allele were obtained by Network 4.6.11 software (Bandelt et al., 1999), including in the analysis one allele of the *O. paniculata* sample, used as an out-group, and one allele for each of the two subspecies *cuspidata* and *cerasiformis*.

The evolutionary history was inferred using the Neighbor-Joining method by MEGA7 software (Kumar et al., 2016). The optimal tree, related to all gene fragments, with the sum of branch length = 0.1183 is shown. The tree was drawn to scale branch lengths at the same units as those of the evolutionary distances used to infer the phylogenetic tree. The evolutionary distances were computed using the Maximum Composite Likelihood method and were given as units of the number of base substitutions per site. The analysis involved 90 nucleotide sequences for the total length of analyzed fragments. The bootstrap analysis was done by using 1,000 replicates. All the others NJ trees, including the analysis of haplotypes for each gene and the *OeACP*1–*OeACP*2 for 56 genotypes, were analyzed by following the same parameters through the same software.

To assess whether signatures of positive selection or demographic processes were present in each of the four candidate loci, three neutrality tests were separately performed (Tajima’s D, Fu and Li’s D and Fu and Li’s F) (Tajima, 1989; Fu and Li, 1993) on exons and introns of gene fragments, by using DnaSP 5.10.01 software (Librado and Rozas, 2009). Tajima’s D statistic is the most widely used neutrality test, while Fu and Li’s D is based on the fact that singletons, i.e., polymorphisms that only affect one individual in a sample, play a special role in the history of a population.

Following the neutrality test results, to verify the presence of any genotype/phenotype correlation for *OeACP* genes, a subset of 56 varieties from the Olive Varietal Collection established in Zagaria (Enna, Italy) was phenotyped for FA composition and fruit weight. Fruit samples were collected from two plants per cultivar, for 2 years and two dates/year. Methods of analysis refer to de la Rosa et al. (2013b). Based on biochemical data, cultivars were grouped into three classes for each trait (fresh fruit weight, percentage of palmitic, palmitoleic and oleic acids) (Supplementary Table S2). The range of each class was established taking into account that the distance between two consecutive classes was higher than the minimal difference within each class (Supplementary Table S3) and according to the data from Oleadb database^7^.

Furthermore, Box and Whisker plots were chosen to graphically show the correlations between genotype and phenotypic traits (Fisher et al., 2016). For each independent SNP or different groups of linkage, Box and Whisker plots were made by comparing distributions of each SNP genotype versus phenotypic trait by means of PAST 3.20 (Hammer et al., 2001). Differences among distributions of phenotypic traits disaggregated by genotypes were tested through Mann–Whitney test conducted through PAST 3.20. The marker effect estimates for each genotypic class (homozygous or heterozygous) for each SNP were estimated using the software package TASSEL 3.0 (Bradbury et al., 2007).

Finally, TASSEL was used to establish genotype/phenotype relationships by using the association analyses with general linear model (GLM) and mixed lineage model (MLM), the latter only for significant correlations by using kinship matrix obtained by the same software. A Q matrix was built by using the Bayesian model-based approach and the “Clustering with linked loci” module, as implemented in BAPS 6.0 (Corander et al., 2008), inferring hidden genetic structure for *OeACP1* and *OeACP2* genes, separately. This software uses a Bayesian model that accounts for the linkage present between polymorphic sites within aligned sequences. For each gene, BAPS carried out a genetic mixture analysis to determine the best partition of the data into K groups. The mixture analysis was run with 100 iterations, the input number of reference genotypes from each group was 10; the input number of iterations for reference genotype was 100, the minimum size of a group was 10. Subsequently, BAPS carried out a genetic admixture analysis to estimate admixture proportions within each genotype, taking into consideration the most likely number of K groups identified previously. TASSEL genotype alignments were first filtered using minimum counts of 56 sequences, with a minimum SNP frequency of 0.05 (minor allele frequencies were excluded due to instability), including all sites for each fragment.

## Results

### Identification of Gene Sequence Polymorphisms

Combined approaches, including cloning and direct sequencing performed by means of locus- and allele-specific primers, allowed us to distinguish all alleles at the four loci on 90 olive cultivars. Nucleotide diversity in the gene fragments is summarized in Table 2. Polymorphisms at each allele, their position on the sequence, amino acid changes and recombination sites are reported in Supplementary Table S4. As expected, nucleotide variations within introns were more numerous and informative than those within exons. In fact, the coding regions, representing a relatively short portion (20.4%) of the total length of the analyzed sequences, carried about 8.7% of variations, exclusively represented by SNPs. Conversely, introns carried most of polymorphisms (91.3%), including SNPs, indels, tandem repeats and simple sequence repeats (SSRs). The 196 total polymorphisms consisted of 174 SNPs, 13 indels, one tandem sequence found in *OeSUT1*, and eight SSRs, the latter exclusively present in the *OeACP2* fragment.

**Table 2:** Nucleotide diversity within the analyzed gene fragments.

Parameter	Locus				Total
		
	*OeACP1*	*OeACP2*	*OeLUS*	*OeSUT1*	
Fragment length interval (bp)	987–990	1,288–1,363	555	729–914	3,559–3,822
Exons length (bp)	93	93	254	340	780
Introns length interval (bp)	894–897	1,195–1,270	301	389–574	2,779–3,042
Total number of polymorphisms	33	127	10	26	196
SNPs on the entire fragment	29	112	10	23	174
SNPs on exons	4	7	2	4	17
Indels	4	7	0	2	13
SSRs	0	8	0	0	8
Frequency of polymorphisms	1/30.0	1/10.7	1/55.5	1/35.2	1/19.5
π	0.013	0.029	0.007	0.009	0.015^∗^
Unlinked polymorphisms	3	15	4	13	35
Linked polymorphisms (linked groups)	30 (3)	104 (5)	6 (2)	13 (2)	153 (12)
True cloned haplotypes	6	13	7	15	41
Calculated haplotypes	11	39	8	26	84


*Number of linked and unlinked polymorphisms is reported, together with that of real and calculated haplotypes. ^∗^Average value.*

Single nucleotide polymorphism frequency was 7.9 times higher than length polymorphisms (indels, repeated sequences, and SSRs). The variation density was one polymorphism every 19.5 bp (only one every 21.9 bp for SNPs), one every 45.9 bp on exons and one every 17 bp on introns. The 17 SNPs detected in coding regions included 11 transitions and six transversions, leading to amino acid changes only in the *OeACP* gene fragments, as detailed below.

#### OeACP1

The analysis of the *OeACP1* allele fragments, with a maximum length of 990 bp, revealed 29 SNPs and four indels, giving a frequency of one polymorphism every 30 bp. A high degree of linkage was observed: on the 33 polymorphisms, only two SNPs at positions 407 and 503 and one indel at position 537 were unlinked, whereas the remaining (90.9%) were grouped in three linkage groups.

#### OeACP2

The *OeACP2* allele fragments, with a maximum length of 1,363 bp, showed higher variability than that found in the other loci. The 127 polymorphisms included 112 SNPs, seven indels and eight SSRs, with a frequency of one polymorphism every 10.7 bp. The *OeACP2* fragments carried insertions and various microsatellite regions, made up of mono-, di-, and tri-nucleotide motifs. As for *OeACP1*, a high linkage degree was observed: 104 polymorphisms (81.9%) were linked in five linkage groups.

#### OeLUS

In this gene fragment (555 bp), ranging from the seventh exon to a portion of the ninth exon, only 10 nucleotide mutations were detected, with a frequency of one every 55.5 bp. Four SNPs were unlinked, whereas the remaining were grouped into two linkage groups.

#### OeSUT1

Overall, the sequence analysis of the *OeSUT1* allele fragments (maximum length of 914 bp) revealed 23 SNPs, two indels of 1 and 19 bp, respectively, and a motif of 166 bp that in some cultivars was repeated twice in a head-tail arrangement, generating a tandem repeated sequence. The alignment of the isolated gene fragments with the *SUT1* sequence of other plant species – i.e., *Populus trichocarpa* (HM749897.1), *Spinacia oleracea* (X67125.1), *Vitis vinifera* (HQ323258.1), or *Verbascum phoeniceum* (FJ797307.1) – showed that the first exon of the olive gene was divided into two portions by a tandem repeated sequence made up of a 166 bp motif. By aligning this sequence with the collection of repetitive DNA elements of Dfam database (Dfam^8^), it was not identified as a part of a transposable element. The frequency of polymorphisms was one every 35.2 bp. Fifty percent of polymorphisms were linked into two linkage groups. No linked SNPs were found among the four gene fragments.

### Characterization of Alleles and Genotypes

The list of alleles found at each locus, along with the corresponding GenBank accession numbers, is reported in Supplementary Table S5. A maximum of two alleles for each variety was observed, as expected in a diploid species as olive. A non-negligible percentage of homozygosity was detected (29.2% in total, apportioned as 41.1% in *OeACP1*, 12.2% in *OeACP2*, 38.9% in *OeLUS*, and 24.4% in *OeSUT1*). The co-segregation analysis of alleles confirmed that all four genes effectively belong to four distinct loci (data not shown).

#### OeACP1

**FIGURE 1:**
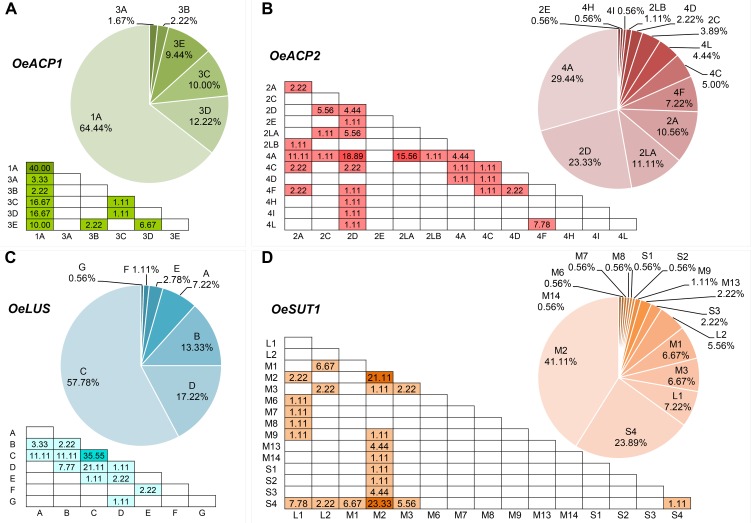
Haplotype (pie charts) and genotype (triangular matrices) frequencies at the four loci **(A–D)** in the 90 cultivars under study. Darker background colors in genotype matrices indicate the most frequent allele combinations.

Six alleles, clustered in two groups, were found for *OeACP1*. The allele named 1A was clearly distinct from the others, due to two deletions at positions 306 and 802 (TGTTGTT and TAG, respectively) and a 12 bp insertion at position 485, joined with 23 linked SNPs. Three out of the four indels were linked. The other alleles belonged to the same group called 3 (3A, 3B, 3C, 3D, 3E), and were distinguished among each other by a few polymorphisms. Three out of four polymorphisms found in exon two generated three amino acid changes (Supplementary Table S4): threonine instead of asparagine in allele 3B (position 217); proline (non-polar and hydrophobic) to arginine (basic, with an amine group in the side chain; position 259) and proline to serine (polar and hydrophilic; position 279), both in 1A allele, the most widely diffused among the analyzed cultivars, reaching 64% of total alleles. Data suggest that alleles did not combine randomly within the analyzed cultivars. In fact, a low number of allele combinations (genotypes) was observed: only 10 out of the 21 possible genotypes (47.6%) were detected. The homozygous 1A represented 40% of total genotypes, followed by 1A-3D and 1A-3C (both 16.7%) (Figure 1A, Table 1, and Supplementary Table S4).

#### OeACP2

This locus showed 13 alleles clustered into three groups. These latter were initially recognized based on distribution of indels and the structure of a highly polymorphic SSR region starting from nucleotide 688 (Supplementary Table S4). Six out of the seven indels were linked with many SNPs and microsatellites, and distinguished the alleles of group 4 (4A, 4C, 4D, 4F, 4H, 4I, 4L) from those forming the second group (2A, 2C, 2D, 2E). The third group included the two longest alleles, 2LA and 2LB, differing from each other by a CTT microsatellite motif (at 688 bp), repeated 18 and 19 times respectively. The seven polymorphisms found in exon 2 gave rise to five amino acid changes: two of them (valine/alanine and isoleucine/valine), determined by SNPs at position 257 and 337, distinguished the alleles 4C and 4F, respectively, and did not cause polarity change. Three out of the seven polymorphisms characterized the shortest alleles of group 2. The first, at position 262, produced aspartic acid (acidic with a carboxylic group in the side chain) instead of asparagine (polar and hydrophilic); the second, at position 292, produced arginine (basic with an amine group in the side chain) instead of glycine (polar and hydrophilic); and the third, at position 318, encoded phenylalanine in place of leucine. Some alleles were widely distributed within varieties, with frequencies exceeding 29% for 4A and 23% for 2D, whereas most of the other alleles were very poorly represented, being found in two (2LB) or just one cultivar (2E, 4H, and 4I). Also in *OeACP2* alleles did not combine randomly. In fact, only 27 out of the possible 91 genotypes (29.7%) were found in our sample set. Out of these, the most frequent genotype was 2D-4A (18.9%), derived from the combination of the most frequent alleles (Figure 1B, Table 1, and Supplementary Table S4).

#### OeLUS

The 10 SNPs detected in the amplified fragment distinguished seven alleles, named from A to G. The two polymorphisms found in exon 8 did not cause any amino acid change. Allele C showed the highest frequency (approximately 58% of total alleles), and only 12 of the possible 28 genotypes were observed, the most common being C–C, shared by the 35% of cultivars (Figure 1C, Table 1, and Supplementary Table S4).

#### OeSUT1

Based on the length polymorphisms, *OeSUT1* alleles were divided into three groups: long (L), medium (M), and small (S). Length differences between S and M alleles were due to a 19 bp indel, whereas L alleles contained a duplication of a 166 bp motif in respect to M alleles. SNPs in the exon sequences did not produce any amino acid change. The M group represented 60% of total alleles and, within this group, M2 showed the highest frequency. The S alleles represented 27% of the total, and the two L alleles only the remaining 13%. No random combinations of *OeSUT1* alleles were observed, in fact, only 22 out of the possible 120 (18.3%) genotypes were identified. The most frequent genotypes were M2-S4 and M2-M2. Taken together, they represented more than 44% of the observed ones (Figure 1D, Table 1, and Supplementary Table S4).

The three neutrality tests applied to gene polymorphisms showed significant high and positive scores for *OeACP1* and *OeACP2* loci compared to those of the other two genes, in particular after the Tajima’s D test (4.11 for *OeACP1* and 3.75 for *OeACP2*, 1.04 for *OeLUS* and 1.81 for *OeSUT1*; *p* < 0.001) (Supplementary Figure S1).

### Linkage Disequilibrium, Intragenic Recombination, and Gene Phylogeny

**FIGURE 2:**
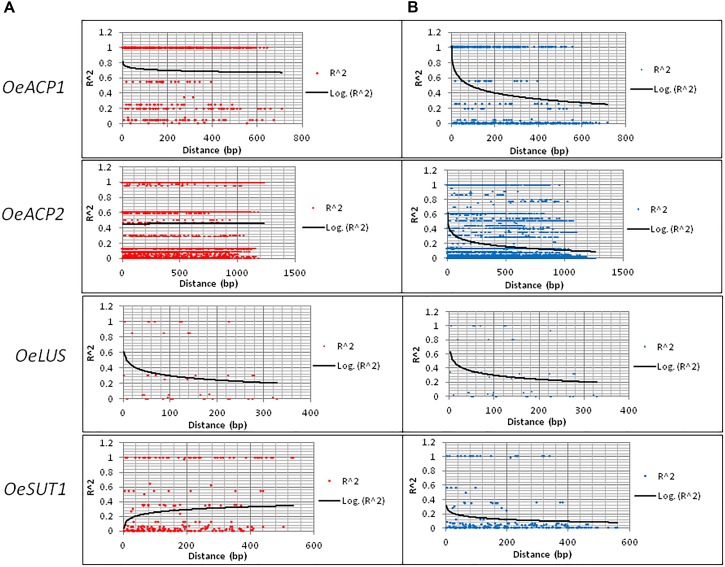
LD decay along the four loci based on real **(A)** and calculated **(B)** haplotypes. LD was measured by calculating *r*^2^ values and computing a non-linear regression line. Real haplotypes refer to those derived from cloning and direct sequencing, while the calculated ones are based on DnaSP analysis of genotypes.

The number of calculated haplotypes detected by the DnaSP program was much higher than that of the true ones identified by cloning and sequencing at all gene fragments, except for *OeLUS* (Table 2). LD analysis at each locus showed a different decay pattern between true and calculated haplotypes except for *OeLUS*. Decay of true haplotypes remained almost constant for the entire fragment length in *ACP* genes and increased in *OeSUT1*, meanwhile decay of true *OeLUS* and all calculated loci significantly decreased (Figure 2).

The analyzed *OeACP1* and *OeLUS* regions were not involved in any recombination event (RE), each representing a ‘haplotype block.’ In *OeACP2*, the IR for calculated haplotypes was 0.252, while the IR obtained with real haplotypes was 0.023, corresponding to a calculated minimum number of 19 recombination events versus the true five ones. The latter were located at 140–147, 147–151, 318–348, 348–470, and 851–1,167 bp. Interestingly, one of them included the last part of the second exon (from 318 to 337 bp). All the other *OeACP2* portions represented haplotype blocks. In *OeSUT1*, the IR value was four times higher in calculated versus true haplotypes, and the only RE was located at 259–566 bp, in the intron region containing the 166 bp repeated motif (Supplementary Table S4).

**FIGURE 3:**
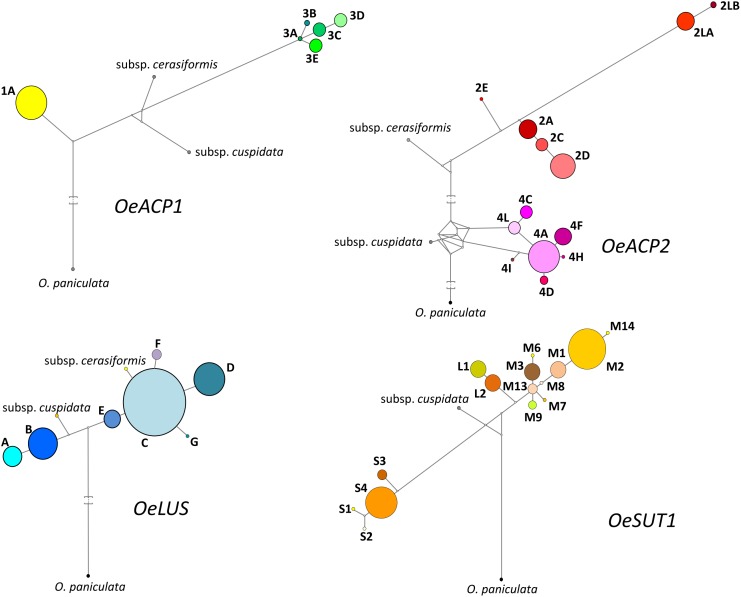
Most parsimonious networks obtained by Network 4.6.11 using SNPs and length polymorphisms for each allele at the four loci. Each haplotype is represented by a colored circle, whose size is related to the frequency within the 90 olive cultivars dataset. Branch length is proportional to the number of mutations. Median vectors (white nodes) correspond to possible extant unsampled or extinct ancestral sequences.

A locus-by-locus phylogeny analysis was assessed, both my NJ analysis based on allele sequences for each gene and by Network results. The most parsimonious networks (based on SNP, indel and SSR polymorphisms), linking the sequences at each locus, are shown in Figure 3 and Supplementary Figure S2. Most alleles at each gene fragment were scattered into well-separated sub-branches. In *OeACP1*, allele 1A was derived from an unknown primordial form, even considering that the two subspecies are located between allele 1A and the alleles of group 3. The ancestral form of all alleles of group 3 seemed to be 3A allele. In *OeACP2*, a complex network was found: the *cuspidata* allele is located as the ancestral form of the entire group 4, whereas the *cerasiformis* allele appeared as the ancestral form of group 2, with the exception of the two longest alleles (2LA and 2LB), whose separation from the other alleles of group 2 was sustained by a bootstrap value of 100% (Supplementary Figures S1, S2). The *OeLUS* alleles were clearly divided into two clades, one including *cuspidata* and A and B alleles, the other including E allele, followed by C allele that gave rise to F, G, D alleles and, interestingly, *cerasiformis* allele was located in this clade. In *OeSUT1*, alleles also grouped into two branches, one including the four S alleles, the other the M and L alleles. The phylogeny of this branch was clearly defined, starting from M13, a sub-branch led to L2 and L1 alleles, whereas a second sub-branch led to all other M alleles, up to M2 and M14. The *cuspidata* allele was located between the short and long-medium alleles. It was not possible to amplify *cerasiformis* allele for *OeSUT1*.

A survey of genotypes at the four loci in all the analyzed cultivars (Table 1), showed that the most widespread alleles of each gene (*OeACP1*-1A, *OeACP2*-4A, *OeLUS*-C, OeSUT1-M2) (Figure 1), were combined together in the 26% of the cultivars. Instead, the allele combinations including *OeACP1*-1A, *OeACP2*-4A, or *OeLUS*-C alleles were shown by 40% of the cultivars considered in this study. At a general extent, no clear relationship was found between the allele combination and geographical distribution.

### Discrimination Power and Genotype/Phenotype Correlations

**FIGURE 4:**
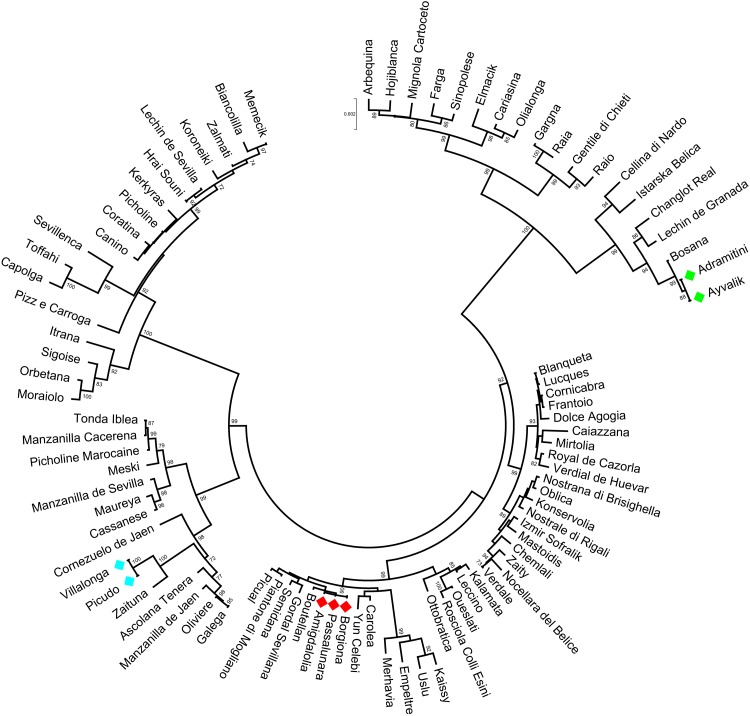
Circle dendrogram obtained by MEGA7 software with Neighbor Joining method showing the relationships among the cultivars analyzed based on total gene sequences. The bootstrap value is showed (cut-off > 70).

The polymorphisms identified in the analyzed gene regions were able to distinguish 92.2% of cultivars, as illustrated in the Neighbor Joining tree, harboring five main clusters (Figure 4). Within three of them, some cultivars, i.e., Borgiona-Passalunara-Amigdalolia and Picudo-Villalonga, were not discriminated by gene markers whereas resulted as distinct genotypes by means of standard SSRs. Instead, the Adramitini-Ayvalik group resulted identical by SSRs (Trujillo et al., 2014) and by the new set of markers. Thirteen varieties were distinguished by using just one polymorphism (SNP or indel) and only two were sufficient to distinguish 32 cultivars (Supplementary Table S6), demonstrating their potential as genotyping markers.

Taking into account the neutrality tests results, indicating that *OeACP* loci were target of artificial selection, the possible correlations between these loci and interesting phenotype traits were evaluated.

**FIGURE 5:**
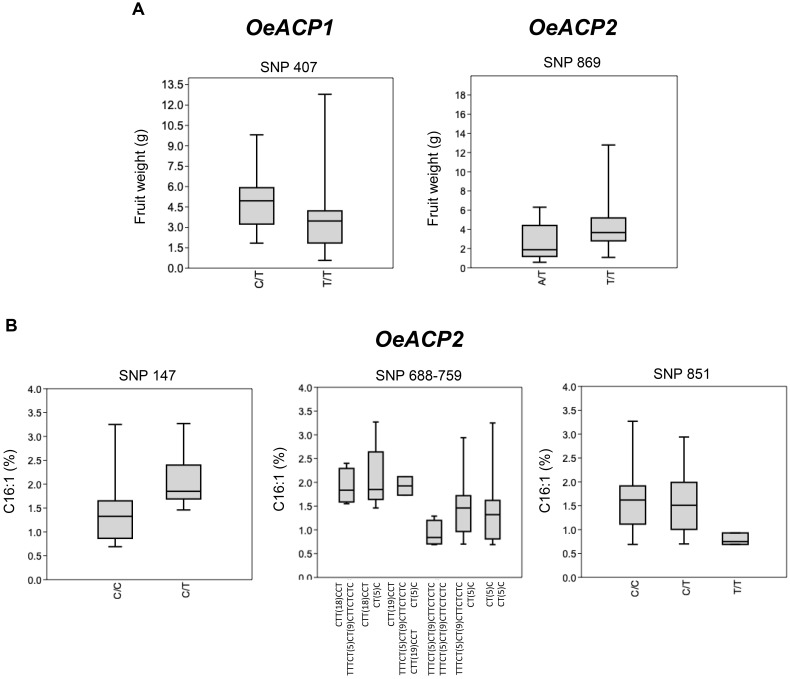
Box and Whisker plot graphs showing the distribution trend of best SNP candidates of *OeACP1* and *OeACP2* versus the studied phenotypic traits. **(A)** Fresh weight; **(B)** palmitoleic acid. Square boxes represent the trend for most samples. These plots display the five-number summary of a set of data: the minimum and maximum, the first quartile, the median and third quartile allowing a comprehensive data comparison. Mann–Whitney test results showing significant differences (*p* < 0.05) among phenotypic distribution disaggregated on the basis of the reported SNPs.

The graphical representation in Box and Whisker plots, performed by merging the *OeACP1* and *OeACP2* loci polymorphisms with the phenotype data of 56 varieties (Figure 5), showed two cases of positive relationships for *OeACP1* SNP 407 (characterizing the genotype combinations with 3D allele) and for *OeACP2* SNP 869 (characterizing the combinations with 4F allele) with fruit weight (Figure 5A), and three cases of SNPs at positions 147, 688, and 851 of *OeACP2*, related to palmitoleic acid (C16:1) percentage (Figure 5B).

When the functional contribution of polymorphisms at each *ACP* locus was evaluated by TASSEL following GLM analysis, significant relationships were confirmed only for the fruit weight trait both for *OeACP1* (*p* = 0.033) and *OeACP2* (*p* = 0.047) (Supplementary Table S7). These two possible associations were also tested by MLM analysis considering also the kinship matrix. Even though the best linked polymorphisms remained the same, both *p*-values increased and went out from the significance (*p* = 0.622 and 0.162, respectively).

An additional Neighbor Joining tree (Supplementary Figure S3) was obtained by merging *OeACP1* and *OeACP2* sequences of 56 cultivars, in order to verify if the cultivars that grouped in the same genetic cluster showed also a relationship with the fruit fresh weight. Almost all cultivars carrying both or only one of the two alleles *OeACP1*-3D and *OeACP2-*4F are included in three clusters (from Carolea to Picual, from Galega to Tonda Iblea, from Itrana to Lechin de Sevilla) and showed a medium-high fruit weight (Supplementary Figure S3), with the exception of the cultivars Ottobratica and Galega.

## Discussion

The analysis of polymorphisms at four olive gene fragments – *OeACP1*, *OeACP2, OeLUS*, and *OeSUT1* – showed high levels of base substitutions, indels and simple sequence repeats. Their detection is particularly important considering the functional role of the analyzed genes, involved in primary metabolism pathways, such as FA synthesis (Tranbarger et al., 2011; Cultrera et al., 2014; De Marchis et al., 2016) and sugar transport (Conde et al., 2007), or, for what concerns lupeol synthase, in the production of nutraceutical compounds (Saleem, 2009). These results appear even more interesting in the light of the results of the neutrality tests, showing significant and positive variations for both *OeACP1* and *OeACP2* loci as a possible consequence of a recent bottleneck and balancing selection followed by a steady varieties cultivation (Maruyama and Fuerst, 1985). Despite this, the olive genome sequencing data of the Leccino and Farga cultivars (Muleo et al., 2012; Cruz et al., 2016) and the assembled genes of a wild olive (Unver et al., 2017), as well as the lack of saturated genetic maps, have not allowed to physically map these genes.

The frequency of total mutations (one every 19.5 bp) and single nucleotide polymorphisms (one every 21.9 bp) was high compared to that found in other plant species, in which SNP frequency was detected by the same sequencing method. The values detected in olive were higher than that observed not only in perennial woody species, such as *Vernicia fordii* (1/576 bp in the *fad2* gene; Ren et al., 2013) or *Vitis vinifera* (1/51 bp for the entire *DXS* gene; Emanuelli et al., 2010), but also in annual herbaceous species, as fescue (1/21 bp for seven gene fragments; Xu et al., 2018), or wheat (1/86 bp in the *TaSnRK2.7* gene; Zhang et al., 2011), that show a higher genetic turnover. In *Eucalyptus camaldulensis*, 1/16 bp was considered the highest SNP frequency of any woody plant species (Kulheim et al., 2009), but within the *OeACP2* gene we found a frequency of 1/12.2 bp on average, considerably exceeding this primacy. High nucleotide diversity at nuclear gene level was already observed in olive cultivars (Sabetta et al., 2013). Interestingly, π diversity value detected within cultivated olive (0.015) resulted higher than that observed in wild olives (0.008; Besnard and El Bakkali, 2014). This difference could be due either to the analyzed genes and/or to plant material (cultivar versus wild type), on which different selection and/or domestication processes could have operated (Besnard et al., 2013, 2018; Diez et al., 2015).

The frequency of indels and microsatellites along the introns of the analyzed fragments of the four loci resulted quite low compared to total polymorphisms (6.6 and 4.1%, respectively). Due to the high SNP frequency and the low indel frequency, their ratio resulted higher than in other plant species: 12.4 against 6.4 in apple (Micheletti et al., 2011), 3.6 in rice (Shen et al., 2004) and 1.8 in common bean (Gaitán-Solís et al., 2008). However, direct comparisons between different sequencing strategies are not straightforward and should be considered with caution (Xu, 2006).

It is interesting to note the peculiarity of *OeSUT1* gene sequences respect to other species. The first exon was interrupted by an intron, not recognized as a part of a transposable element (Carrier et al., 2012). Within the intron, a recombination region containing a 166 bp repeated motif, typical of the long alleles of this gene, and numerous nucleotide polymorphisms distinguishing M and S alleles were found. As shown in other out-crossing plant species, a high number of mutation points in regions containing indels, probably deriving from errors during meiosis (Hollister et al., 2010), could be useful to study gene evolution (Mu et al., 2011).

The direct sequencing approach applied in this study, although slow, expensive and labor intensive in comparison to next-generation sequencing, has guaranteed a high accuracy and a reliable estimate of the genetic variability (Ganal et al., 2009).

Despite the high level of variation, the analysis of LD along the four fragments revealed a strong degree of association among sites, even with an opposite trend, and shared between coding regions and introns. The allele-sequencing approach applied to the four gene fragments also allowed us to estimate IR for real haplotypes and to make a comparison with the calculated haplotypes. IR events in true haplotypes, four times lower than the calculated ones, occurred only in *OeACP2* and *OeSUT*, as already evidenced by the comparison of the LD decay curves between real and calculated haplotypes. The portions of these genes not involved in recombination events, as well as the entire fragments of *OeACP1* and *OeLUS*, represented ‘haplotypes blocks.’ As reported first in the human genome (Gabriel et al., 2002; Cardon and Abecasis, 2003), but also in plants such as maize (Rafalski and Morgante, 2004), rice (Yonemaru et al., 2012), or wheat (Hao et al., 2012), the haplotype blocks are regions with little evidence of recombination and, consequently, only few haplotypes can be observed, as in the case of the analyzed olive genes. A similar result was previously obtained in the same species, when only one recombined haplotype was observed by investigating the sequence variation at five nuclear single-copy genes in native and invasive olive accessions (Besnard and El Bakkali, 2014).

Furthermore, in *OeACP2* and *OeSUT1* the recombination sites were detected both in the intron and exon regions. This result contrasts with what observed in the *DXS* gene of grape, in which most of the haplotype blocks were located within introns (Emanuelli et al., 2010).

In our case, a low number of real haplotypes and consequent low haplotype combinations was detected, because recombination events, *per se* factors of differentiation (Nachman and Payseur, 2012; Renaut et al., 2013), occurred essentially within few alleles, lowering the probability to obtain a consistent number of new genotypes.

As already stated, the observed genotypes were very low, being just over a quarter of the potential ones (27.3%). Several hypotheses can be conceived to explain this evidence: (i) a different level of ancestrality may have affected the chance of combination; (ii) the complex varietal diversification process (Hamman-Khalifa et al., 2007; Haouane et al., 2011; Belaj et al., 2012; Besnard et al., 2013; Diez et al., 2015) could have recently shaped the occurrence of haplotypes therefore inducing some biases in gene combinations; (iii) the analyzed genotypes are cultivated varieties of a very long living species that has undergone an artificial selection along 1000s of years; it is likely that the selection made by growers and the adaptation to different environmental conditions, as well as the fitness of the different combinations, may have prevented certain allelic combinations to occur; (iv) inter-incompatibility among varieties (Saumitou-Laprade et al., 2017) might have favored certain allele combinations to the detriment of others, or some alleles might have been established over others because certain combinations resulted poorly competitive or lethal. While in natural populations, evolution is characterized by the clinal variation of allele frequencies, due to local adaptive processes (Maynard and Haigh, 2007; Chen et al., 2012), in non-natural populations such as cultivated olives, genes that control the phenotypic traits have been fixed through the artificial selection and vegetative propagation, thus reducing the possibility of recombination. Although the causative mutations under selection are unknown, their fixation could have led, in the proximity of the selected loci, to a decrease of genetic diversity through a hitchhiking effect (Palaisa et al., 2004; Cutter and Payseur, 2013).

The use of single-copy genes to reconstruct phylogenies of the olive complex, has been limited by the high allele diversity, coupled to numerous ancient admixture events, because very different topologies may be supported by different loci (Wendel and Doyle, 1998; Besnard and El Bakkali, 2014). In this research, phylogenetic analyses showed that, for three out of four analyzed genes, allele fragments were clustered into two or three groups together with the alleles of the analyzed subspecies. The lack of direct ancestral forms connecting the haplotypes of cultivated olives with those of *O. paniculata* species, was probably due to the existence of unsampled or extinct ancestral sequences. The high affinity between the allele clusters showed that the different allele forms derived from each other through single or multiple nucleotide mutations. Moreover, the relationships established in our material may be considered as an evidence of introgression of alleles in cultivated olives from other subspecies. Interesting is the case of *OeACP1*-1A allele, highly widespread among cultivars of different geographical origin, that appeared before all other alleles, including those carried by different subspecies, and for which a different introgression process could be hypothesized. In *OeACP2*, a close affinity with subsp. *cuspidata* can be deduced for alleles of group 4, whereas group 2 alleles derived from subsp. *cerasiformis*. A similar frame can be seen in *OeLUS*, in which the two subspecies are distinctly linked to different allele groups. It is interesting to highlight that *cerasiformis* subspecies is characteristic to the extreme western side of Mediterranean, while *cuspidata* subspecies spreads from north-east and south of Africa to the south of Asia, two completely different ways of diffusion.

As interpreted for the distribution of lupeol synthase alleles in wild Mediterranean olives (Besnard and El Bakkali, 2014), the recurrent admixtures among gene pools occurred during the varietal diversification process in olive, probably led to the present geographical dispersion of the alleles studied.

This also explains why in the set of cultivars analyzed, which included the main commercial ones, it was not possible to group the varieties according to their geographical origin. Despite this, some alleles or allelic associations have been reasonably regionalized, see the French cultivars and the case of *OeACP2*-2L, which is more present in the cultivars of the West-Central Mediterranean and characterizes a large number of Spanish cultivars. The same result was obtained by specific Neighbor-Joining analysis.

Due to the strong association, only 21% on total polymorphisms resulted informative, but these were able to discriminate a vast majority of the analyzed cultivars. Some polymorphisms (e.g., in *OeACP2*) were specific to single varieties (i.e., Arbequina, Rosciola Colli Esini, Zaity), thus representing powerful markers, exploitable not only for genotyping purposes but also for olive oil DNA testing, parentage analysis and association mapping studies.

This low recombination level, together with a very high and significant values of Tajima test, encouraged us to investigate the occurrence of any genotypic/phenotypic correlation. Box and Whisker plots showed a good correlation between fruit weight and two specific polymorphisms, the *OeACP1* SNP at position 407 characterizing allele 3D, and *OeACP2* SNP at position 869, characterizing allele 4F. The 3D allele was present in 26.8% of cultivars, six of them representing the most outstanding large-fruited table olive varieties (e.g., Gordal Sevillana, Kalamata, and Ascolana Tenera), and other six double-purpose (oil and table olives) varieties (e.g., Biancolilla, Hojiblanca, and Amigdalolia). Box plot graphs highlighted also three SNPs in *OeACP2* sequences as significantly related to the percentage of palmitoleic acid. When each relationship was tested by TASSEL, only the correlation between fruit weight and both SNPs in 4F and 3D alleles remained within the significance range, when using GLM analysis. Box and Whisker plots, produced on the basis of the allele estimates output provided by TASSEL, underlined that when the estimate values were >0.5 the effect of allele’s genotypic class clearly emerged. In particular, homozygous genotypes were associated with lower phenotypic values.

The lack of strong marker/phenotype correlation may be due to numerous factors. Fruit weight and FAs content are quantitative traits presumably controlled by multiple loci and the effect of one over the others is still totally unknown. Acyl carrier protein is a cofactor and a structural protein that serves for the plastidial condensation of the acyl chains C16, but it represents a member of the FAs synthesis chain that involves different actors (Li et al., 2016). *ACP* genes are characterized, in the upstream and downstream sequences, by regulatory sequences that could play a major role in affecting the trait and, in an oil crop as olive, genes involved in oil accumulation may have undergone to a strong selective pressure, shaping the distribution, frequency and combination of the different alleles. Further functional genomic studies are needed to validate these SNPs as effective putative causal polymorphisms.

## Conclusion

This research showed how an in-depth gene sequence analysis may help to clarify important issues related to allele variation and apportioning of variability in a perennial cultivated species.

The polymorphisms found could be applied for the discrimination and evaluation of the olive genetic resources, in studies on the evolution and domestication of the crop, or as a starting point for further functional genomic experiments.

## Author Contributions

NC, LB, MC, RM, and VS conceived and designed the experiments. NC, VS, FA, RM, SM, and CR performed the experiments. RM, LL, NC, LB, and FA analyzed the data. LB and MC contributed reagents, materials, and analysis tools. LB, NC, RM, LL, SM, FA, and MC wrote the manuscript. All the authors read and approved the manuscript.

## Conflict of Interest Statement

The authors declare that the research was conducted in the absence of any commercial or financial relationships that could be construed as a potential conflict of interest.

## Footnotes

1 www.mbio.ncsu.edu/BioEdit/bioedit.html

2 www.geneious.com

3 http://denovo.cnag.cat/genomes/olive/

4 http://www.oleagenome.org/

5 https://blast.ncbi.nlm.nih.gov/

6 http://olivegenome.org/

7 www.oleadb.it

8 www.dfam.org

## Supplementary Material

Figure S1 Values of three neutrality tests (Tajima’s D, Fu and Li’s D^∗^, and Fu and Li’s F^∗^) performed on the four loci in the 90 cultivars studied. Tests’ significance is reported (^∗∗^*p* < 0.05; ^∗∗∗^*p* < 0.001).

Figure S2 The Neighbor Joining dendrogram showing the relationship among the cultivars analyzed based on the polymorphisms of each allele for each gene. The bootstrap value is showed (cut-off > 60).

Figure S3 Circle dendrogram obtained by MEGA7 software with Neighbor Joining method showing the relationships among the cultivars analyzed based on *OeACP1* and *OeACP2* sequences. The bootstrap value is showed (cut-off > 70).

Table S1 Specific primers used to amplify all alleles at each locus in the analyzed 90 cultivars.

Table S2 Fresh fruit weight and percentages of palmitic (C16:0), palmitoleic (C16:1), and oleic (C18:1) acid in the fruit for 56 olive cultivars.

Table S3 Ranges for each class for fresh fruit weight and percentages of palmitic (C16:0), palmitoleic (C16:1), and oleic (C18:1) acid are reported.

Table S4 Haplotypes obtained at the four gene fragments in the 90 cultivars, with SNPs, indels and SSRs detected. No color refers to unlinked polymorphisms, different colors refer to different groups of linkage. Purple boxes indicate the position of recombination sites. Amino acid changes are reported.

Table S5 List of the haplotypes at each locus and corresponding accession numbers in GenBank.

Table S6 Minimum number of polymorphisms able to discriminate the 90 cultivars analyzed.

Table S7 Summary of general linear model (GLM) and mixed linear model (MLM) results. For each phenotype, *p*-value, type of polymorphism, polymorphism position, and genotype occurrence are reported.
