# Supplementary figures and images for: High Levels of Variation Within Gene Sequences of Olea europaea L
Source: Front Plant Sci. 2019 Jan 8;9:1932. doi: 10.3389/fpls.2018.01932 (PMC6331486; doi:10.3389/fpls.2018.01932)

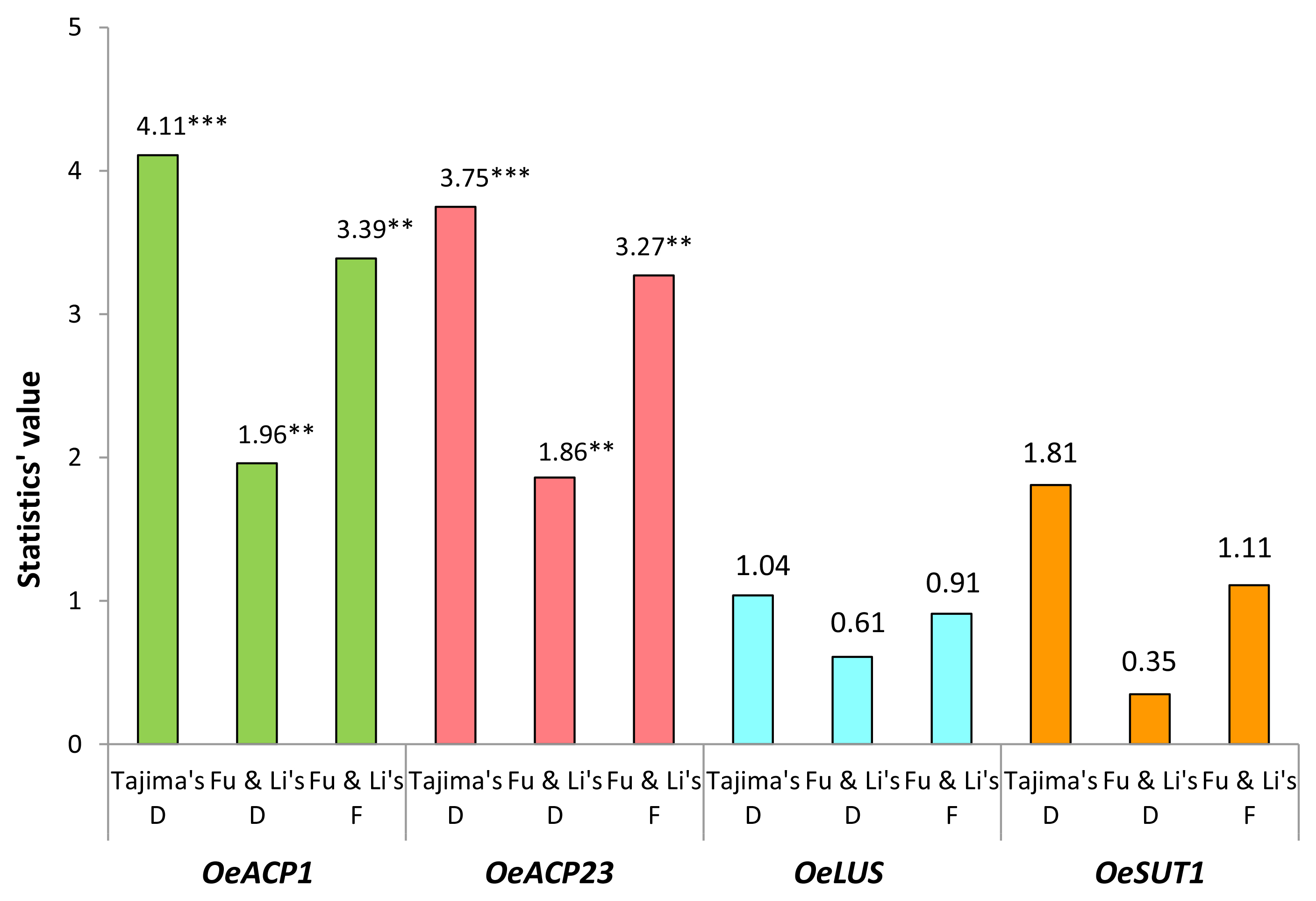

Supplement: Figure S1 — Values of three neutrality tests (Tajima’s D, Fu and Li’s D∗, and Fu and Li’s F∗) performed on the four loci in the 90 cultivars studied. Tests’ significance is reported (∗∗p < 0.05; ∗∗∗p < 0.001). [file Image_1.JPEG]

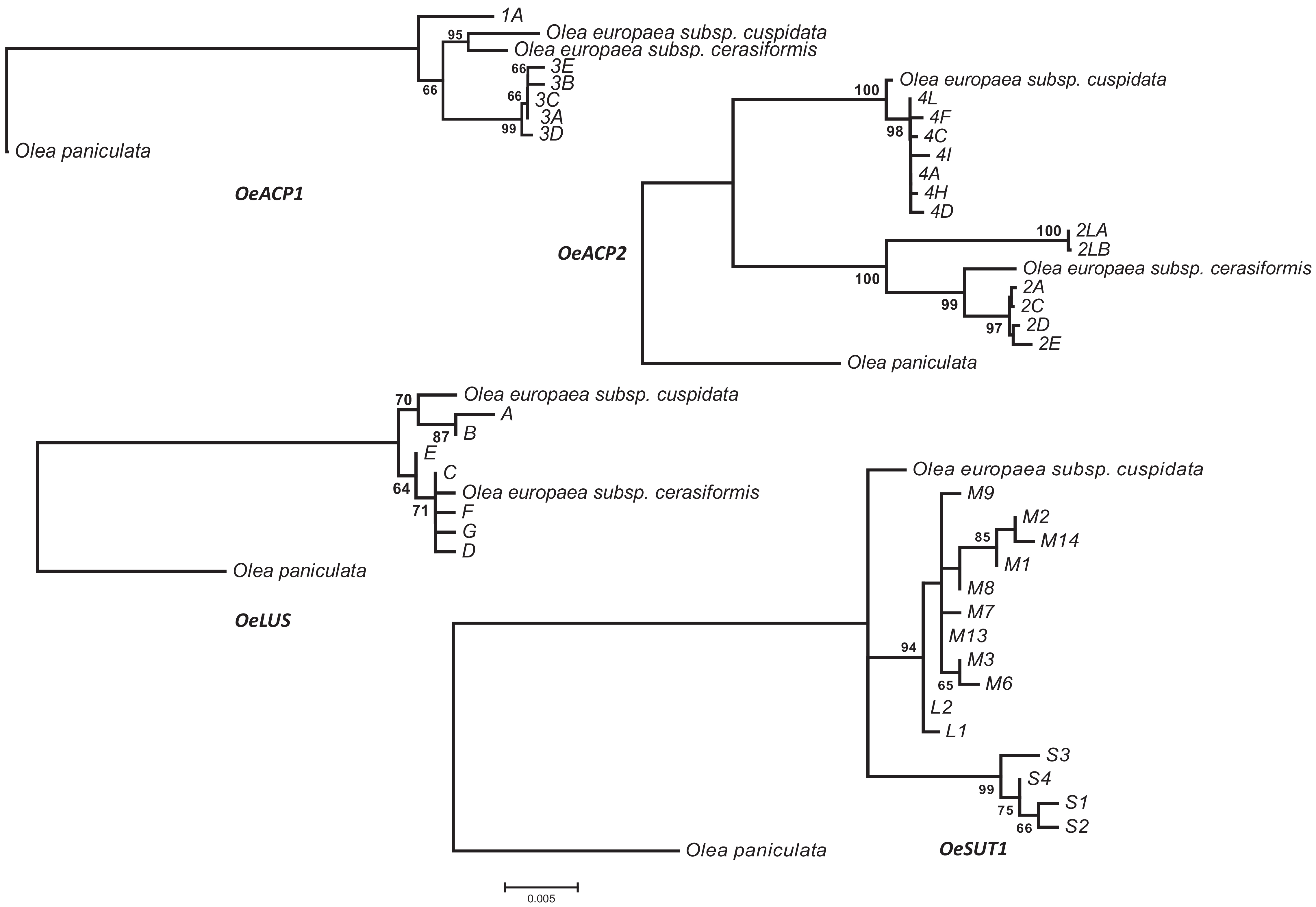

Supplement: Figure S2 — The Neighbor Joining dendrogram showing the relationship among the cultivars analyzed based on the polymorphisms of each allele for each gene. The bootstrap value is showed (cut-off > 60). [file Image_2.JPEG]

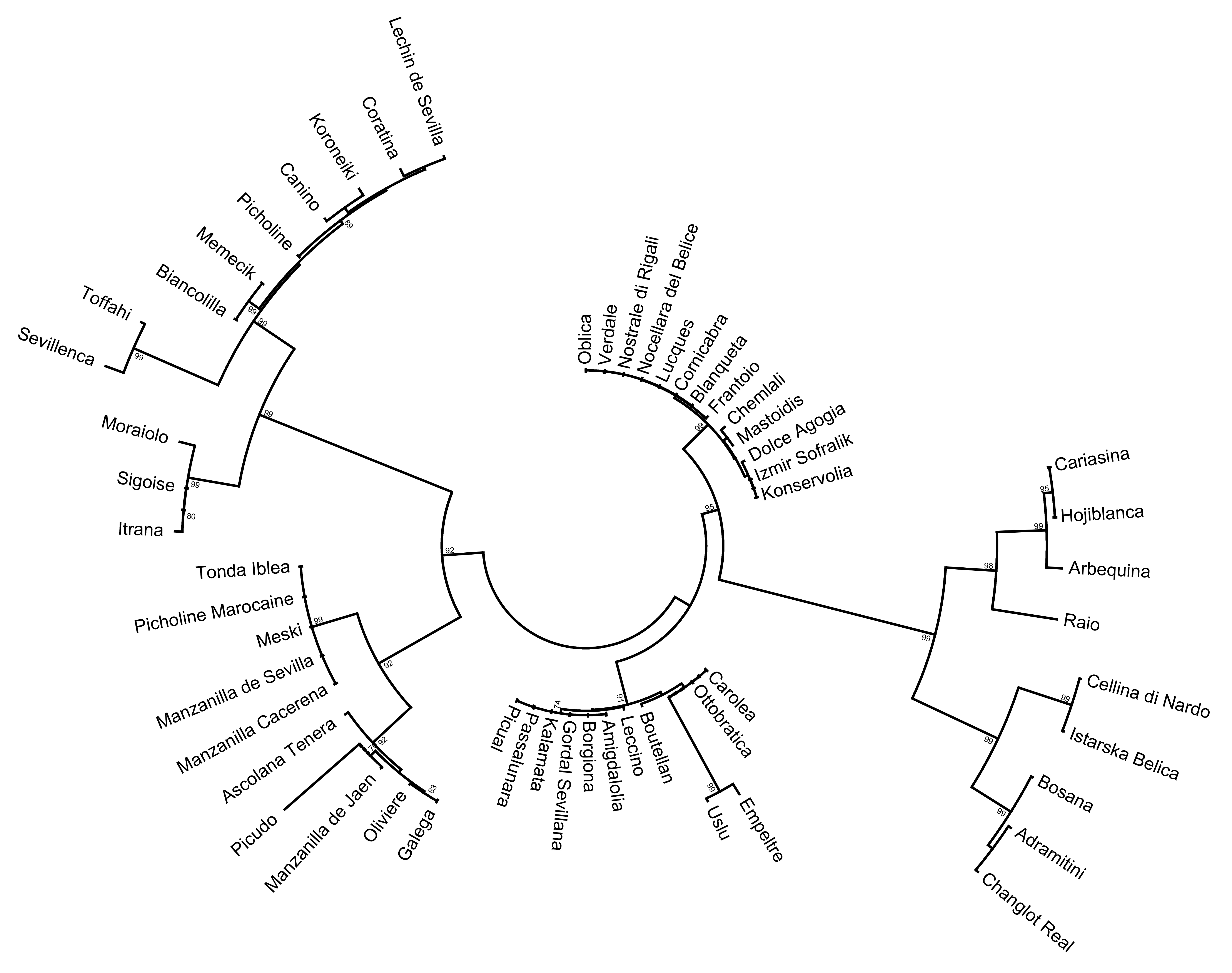

Supplement: Figure S3 — Circle dendrogram obtained by MEGA7 software with Neighbor Joining method showing the relationships among the cultivars analyzed based on OeACP1 and OeACP2 sequences. The bootstrap value is showed (cut-off > 70). [file Image_3.JPEG]
