# Supplementary material for: High Levels of Variation Within Gene Sequences of Olea europaea L
Source: Front Plant Sci. 2019 Jan 8;9:1932. doi: 10.3389/fpls.2018.01932 (PMC6331486; doi:10.3389/fpls.2018.01932)
Supplement: Table S1 — Specific primers used to amplify all alleles at each locus in the analyzed 90 cultivars. [file Table_1.DOC]

**Supplementary Table S1 |** Specific primers used to amplify all alleles at each locus in the analyzed 90 cultivars.

| **Locus** | **Primer forward** | **Primer reverse** |
| --- | --- | --- |
| *OeACP1* | TTTTCGATGTTATTTTGTTCTTGA | AATATTGAAAAACAGATCGATTGAC |
| *OeACP2* | TTCTTAAACGATTTTATGTGTTCCT | TTGTTAATGCTGCACATATCCAT |
| *OeLUS* | GTTCTAACTCGATGGCCGTTTTCTA | TCCCACATTTGACACCCAAA |
| *OeSUT1* | GTTTGCCGCAGATATTGGTC | AAATATTGAGAGGAAAAAGCAGCTC |
