# Supplementary material for: High Levels of Variation Within Gene Sequences of Olea europaea L
Source: Front Plant Sci. 2019 Jan 8;9:1932. doi: 10.3389/fpls.2018.01932 (PMC6331486; doi:10.3389/fpls.2018.01932)
Supplement: Table S2 — Fresh fruit weight and percentages of palmitic (C16:0), palmitoleic (C16:1), and oleic (C18:1) acid in the fruit for 56 olive cultivars. [file Table_2.DOCX]

**Supplementary Table S2 |** Fresh fruit weight and percentages of palmitic (C16:0), palmitoleic (C16:1) and oleic (C18:1) acid in the fruit for 56 olive cultivars.

| **Cultivar** | **Fresh fruit weight (g)** | **% C16:0** | **% C16:1** | **% C18:1** |
| --- | --- | --- | --- | --- |
| Adramitini | 3.47 | 16.17 | 1.55 | 73.02 |
| Amigdalolia | 5.19 | 13.18 | 1.15 | 71.80 |
| Arbequina | 1.14 | 14.09 | 1.29 | 76.71 |
| Ascolana Tenera | 9.81 | 14.65 | 1.98 | 73.48 |
| Biancolilla | 3.40 | 13.01 | 1.01 | 75.95 |
| Blanqueta | 1.08 | 24.28 | 2.94 | 41.82 |
| Borgiona | 3.31 | 11.84 | 0.75 | 76.76 |
| Bosana | 2.89 | 15.44 | 1.97 | 70.80 |
| Boutellan | 5.79 | 18.67 | 1.44 | 48.96 |
| Canino | 1.82 | 14.26 | 1.77 | 75.89 |
| Cariasina | 3.87 | 12.32 | 0.92 | 75.28 |
| Carolea | 3.67 | 13.10 | 0.83 | 76.87 |
| Cellina di Nardo | 1.70 | 14.50 | 1.70 | 71.40 |
| Changlot Real | 6.61 | 13.86 | 2.12 | 73.66 |
| Chemlali | 0.60 | 15.69 | 1.46 | 71.60 |
| Coratina | 3.94 | 11.89 | 0.73 | 80.91 |
| Cornicabra | 3.65 | 15.83 | 2.05 | 74.52 |
| Dolce Agogia | 1.89 | 11.90 | 1.31 | 76.25 |
| Empeltre | 4.08 | 11.97 | 2.08 | 80.32 |
| Frantoio | 2.82 | 13.52 | 0.75 | 79.47 |
| Galega | 1.77 | 12.98 | 1.85 | 80.30 |
| Gordal Sevillana | 7.10 | 14.04 | 1.62 | 74.52 |
| Hojiblanca | 2.64 | 12.08 | 0.93 | 79.20 |
| Istarska Belica | 3.36 | 15.13 | 2.40 | 73.05 |
| Itrana | 5.03 | 17.56 | 1.48 | 61.22 |
| Izmir Sofralik | 3.96 | 11.05 | 0.92 | 81.77 |
| Kalamata | 4.72 | 10.47 | 1.47 | 80.98 |
| Konservolia | 4.95 | 12.05 | 2.06 | 79.48 |
| Koroneiki | 0.57 | 10.65 | 0.81 | 84.00 |
| Leccino | 2.63 | 16.68 | 1.65 | 75.89 |
| Lechin de Sevilla | 2.80 | 15.93 | 1.62 | 73.79 |
| Lucques | 4.08 | 19.70 | 2.45 | 62.31 |
| Manzanilla Cacerena | 6.29 | 12.70 | 1.73 | 80.52 |
| Manzanilla de Jaen | 3.81 | 17.13 | 3.27 | 68.40 |
| Manzanilla de Sevilla | 3.58 | 13.10 | 2.74 | 77.32 |
| Mastoidis | 1.75 | 12.31 | 1.01 | 75.31 |
| Memecik | 6.31 | 16.04 | 1.32 | 68.15 |
| Meski | 5.13 | 11.57 | 1.84 | 71.27 |
| Moraiolo | 1.88 | 16.11 | 0.91 | 74.75 |
| Nocellara del Belice | 4.35 | 15.86 | 1.70 | 64.04 |
| Nostrale di Rigali | 3.92 | 11.82 | 0.70 | 72.6 |
| Oblica | 5.25 | 12.41 | 0.72 | 78.84 |
| Oliviere | 5.27 | 16.48 | 2.54 | 73.30 |
| Ottobratica | 1.36 | 12.90 | 0.94 | 78.65 |
| Passalunara | 5.70 | 12.17 | 0.99 | 79.90 |
| Picholine | 3.03 | 14.81 | 3.25 | 73.39 |
| Picholine Marocaine | 2.61 | 8.98 | 1.69 | 84.12 |
| Picual | 1.84 | 12.05 | 1.65 | 80.16 |
| Picudo | 3.04 | 14.25 | 1.46 | 74.54 |
| Raio | 1.79 | 11.56 | 0.69 | 77.82 |
| Sevillenca | 3.78 | 16.97 | 0.85 | 58.27 |
| Sigoise | 3.62 | 11.75 | 0.69 | 68.74 |
| Toffahi | 12.79 | 17.80 | 1.33 | 59.68 |
| Tonda Iblea | 5.45 | 15.63 | 1.59 | 72.12 |
| Uslu | 5.51 | 12.42 | 1.41 | 68.11 |
| Verdale | 3.16 | 18.54 | 1.74 | 58.12 |
