# Supplementary material for: High Levels of Variation Within Gene Sequences of Olea europaea L
Source: Front Plant Sci. 2019 Jan 8;9:1932. doi: 10.3389/fpls.2018.01932 (PMC6331486; doi:10.3389/fpls.2018.01932)
Supplement: Table S3 — Ranges for each class for fresh fruit weight and percentages of palmitic (C16:0), palmitoleic (C16:1), and oleic (C18:1) acid are reported. [file Table_3.doc]

**Supplementary Table S3 |** Ranges for each class for fresh fruit weight and percentages of palmitic (C16:0), palmitoleic (C16:1) and oleic (C18:1) acid are reported.

| **Classes** | **Phenotype traits** | | | |
| --- | --- | --- | --- | --- |
|  | **Fresh fruit weight (g)** | **% C 16:0** | **% C 16:1** | **% C 18:1** |
| **Low** | 0.57 – 1.89 | 8.98 – 12.42 | 0.69 – 1.01 | 41.82 – 68.74 |
| **Medium** | 2.61 – 4.08 | 12.70 – 14.81 | 1.29 – 1.85 | 70.80 – 73.79 |
| **High** | 4.35 -12.79 | 15.13 – 24.28 | 1.97 – 3.27 | 74.52 – 84.12 |
