# Supplementary material for: High Levels of Variation Within Gene Sequences of Olea europaea L
Source: Front Plant Sci. 2019 Jan 8;9:1932. doi: 10.3389/fpls.2018.01932 (PMC6331486; doi:10.3389/fpls.2018.01932)
Supplement: Table S5 — List of the haplotypes at each locus and corresponding accession numbers in GenBank. [file Table_5.DOC]

**Supplementary Table S5 |** List of the haplotypes at each locus and corresponding accession numbers in GenBank.

| **Locus** | **Haplotype** | **Accession number** |
| --- | --- | --- |
| *OeACP1* | 1A | JN656261.1 |
|  | 3A | JN656258.1 |
|  | 3B | JN656259.1 |
|  | 3C | JN656262.1 |
|  | 3D | JN656263.1 |
|  | 3E | JN656261.1 |
| *OeACP2* | 2LA | JN656264.1 |
|  | 2LB | JN656265.1 |
|  | 2A | JN656267.1 |
|  | 2C | JN656268.1 |
|  | 2D | JN656266.1 |
|  | 2E | JN656269.1 |
|  | 4A | JN656272.1 |
|  | 4C | JN656275.1 |
|  | 4D | JN656273.1 |
|  | 4F | JN656270.1 |
|  | 4I | JN656271.1 |
|  | 4H | JN656274.1 |
|  | 4L | JN656276.1 |
| *OeLUS* | A | JN656235.1 |
|  | B | JN656236.1 |
|  | C | JN656237.1 |
|  | D | JN656238.1 |
|  | E | JN656239.1 |
|  | F | JN656240.1 |
|  | G | JN656241.1 |
| *OeSUT1* | L1 | JN656242.1 |
|  | L2 | JN656243.1 |
|  | M1 | JN656244.1 |
|  | M2 | JN656245.1 |
|  | M3 | JN656246.1 |
|  | M6 | JN656247.1 |
|  | M7 | JN656248.1 |
|  | M8 | JN656249.1 |
|  | M9 | JN656250.1 |
|  | M13 | JN656251.1 |
|  | M14 | JN656252.1 |
|  | S1 | JN656254.1 |
|  | S2 | JN656255.1 |
|  | S3 | JN656256.1 |
|  | S4 | JN656257.1 |
