# Supplementary material for: High Levels of Variation Within Gene Sequences of Olea europaea L
Source: Front Plant Sci. 2019 Jan 8;9:1932. doi: 10.3389/fpls.2018.01932 (PMC6331486; doi:10.3389/fpls.2018.01932)
Supplement: Table S6 — Minimum number of polymorphisms able to discriminate the 90 cultivars analyzed. [file Table_6.DOCX]

**Supplementary Table S6 |** Minimum number of polymorphisms able to discriminate the 90 cultivars analyzed.

| **SAMPLE** | **LOCUS** | **BASE*** | **LOCUS** | **BASE** | **LOCUS** | **BASE** | **LOCUS** | **BASE** | **LOCUS** | **BASE** |
| --- | --- | --- | --- | --- | --- | --- | --- | --- | --- | --- |
| ARBEQUINA | OeACP2-591 | **S** |  |  |  |  |  |  |  |  |
| CAIAZZANA | OeSUT1-79 | **T** |  |  |  |  |  |  |  |  |
| CAPOLGA | OeLUS-347 | **C** |  |  |  |  |  |  |  |  |
| ELMACIK | OeSUT1-590 | **A** |  |  |  |  |  |  |  |  |
| LECHINSEVILLA | OeACP2-257 | **C** |  |  |  |  |  |  |  |  |
| MANZANILLACACERENA | OeACP2-598C | **A** |  |  |  |  |  |  |  |  |
| MASTOIDIS | OeLUS-450 | **M** |  |  |  |  |  |  |  |  |
| OLIALONGA | OeSUT1-238 | **W** |  |  |  |  |  |  |  |  |
| ROSCIOLACOLLIESINI | OeACP2-1296 | **S** |  |  |  |  |  |  |  |  |
| SIGOISE | OeSUT1-207 | **Y** |  |  |  |  |  |  |  |  |
| TOFFHAI | OeSUT1-640 | **Y** |  |  |  |  |  |  |  |  |
| YUNCELEBI | OeSUT1-304 | **W** |  |  |  |  |  |  |  |  |
| ZAITY | OeACP2-372 | **Y** |  |  |  |  |  |  |  |  |
| BIANCOLILLA | OeSUT1-271 | **R** | OeLUS-259 | **G** |  |  |  |  |  |  |
| BOSANA | OeACP2-688F | **C/DEL** | OeACP1-537 | **T/DEL** |  |  |  |  |  |  |
| CANINO | OeACP2-257 | **Y** | OeACP2-239B | **T/DEL** |  |  |  |  |  |  |
| CARIASINA | OeACP2-1120B | **T** | OeSUT1-176 | **T** |  |  |  |  |  |  |
| CELLINA | OeACP2-688F | **C/DEL** | OeSUT1-176 | **G** |  |  |  |  |  |  |
| CHANGLOTREAL | OeACP2-688F | **C/DEL** | OeACP2-348 | **G** |  |  |  |  |  |  |
| CHEMLALI | OeSUT1-643 | **T** | OeACP2-688A | **T/DEL** |  |  |  |  |  |  |
| CORATINA | OeACP2-103 | **S** | OeACP2-257 | **Y** |  |  |  |  |  |  |
| DOLCEAGOGIA | OeLUS-174 | **Y** | OeACP2-257 | **Y** |  |  |  |  |  |  |
| EMPELTRE | OeACP1-217 | **M** | OeSUT1-566 | **Y** |  |  |  |  |  |  |
| FRANTOIO | OeACP2-688A | **T/DEL** | OeLUS-259 | **Y** |  |  |  |  |  |  |
| GARGNA | OeACP2-1120B | **T** | OeACP1-245 | **A** |  |  |  |  |  |  |
| GORDAL | OeSUT1-590 | **R** | OeLUS-277 | **K** |  |  |  |  |  |  |
| HOJIBLANCA | OeSUT1-271 | **R** | OeACP2-28 | **C** |  |  |  |  |  |  |
| KAISSY | OeSUT1-643 | **T** | OeACP1-217 | **M** |  |  |  |  |  |  |
| KALAMATA | OeSUT1-271 | **R** | OeACP2-1167 | **R** |  |  |  |  |  |  |
| KERKIRAS | OeLUS-174 | **Y** | OeACP2-257 | **T** |  |  |  |  |  |  |
| LECHINGRANADA | OeACP2-688F | **C/DEL** | OeSUT1-643 | **c** |  |  |  |  |  |  |
| MAUREYA | OeACP2-688A | **C/DEL** | OeLUS-122 | **Y** |  |  |  |  |  |  |
| MEMECIK | OeSUT1-271 | **R** | OeLUS-259 | **R** |  |  |  |  |  |  |
| MIGNOLA | OeACP2-851 | **T** | OeLUS-122 | **Y** |  |  |  |  |  |  |
| MIRTOLIA | OeACP2-688A | **T/DEL** | OeSUT1-243 | **Y** |  |  |  |  |  |  |
| NOCELLARABELICE | OeLUS-259 | **G** | OeACP1-245 | **A** |  |  |  |  |  |  |
| OBLICA | OeSUT1-566 | **C** | OeACP2-1120B | **T/DEL** |  |  |  |  |  |  |
| OUSLATI | OeACP2-688A | **T/DEL** | OeACP1-245 | **M** |  |  |  |  |  |  |
| PICUAL | OeSUT1-590 | **R** | OeSUT1-176 | **T** |  |  |  |  |  |  |
| PIZZECARROGA | OeSUT1-643 | **Y** | OeACP1-181 | **M** |  |  |  |  |  |  |
| RAIO | OeACP2-851 | **T** | OeSUT1-643 | **Y** |  |  |  |  |  |  |
| SEVILLENCA | OeACP2-239B | **T/DEL** | OeACP1-181 | **M** |  |  |  |  |  |  |
| TONDAIBLEA | OeSUT1-643 | **T** | OeACP2-688A | **C/DEL** |  |  |  |  |  |  |
| USLU | OeACP1-306-312 | **T** | OeSUT1-243 | **Y** |  |  |  |  |  |  |
| VERDIALHUEVAR | OeSUT1-176 | **G** | OeLUS-277 | **K** |  |  |  |  |  |  |
| ADRAMITINI | OeACP2-688F | **C/DEL** | OeLUS-442 | **G** | OeACP1-537 | **T** |  |  |  |  |
| ASCOLANATENERA | OeACP1-407 | **Y** | OeACP2-108 | **W** | OeACP1-181 | **C** |  |  |  |  |
| AYVALIK | OeACP2-688F | **C/DEL** | OeLUS-442 | **G** | OeACP1-537 | **T** |  |  |  |  |
| CASSANESE | OeACP2-688A | **C/DEL** | OeLUS-277 | **K** | OeSUT1-643 | **T/DEL** |  |  |  |  |
| CORNEZUELOJAEN | OeACP2-688A | **C/DEL** | OeLUS-277 | **K** | OeACP1-181 | **M** |  |  |  |  |
| FARGA | OeSUT1-176 | **G** | OeACP2-28 | **C** | OeACP1-181 | **M** |  |  |  |  |
| GALEGA | OeSUT1-176 | **G** | OeLUS-442 | **G** | OeACP1-537 | **T** |  |  |  |  |
| GENTILECHIETI | OeACP2-851 | **T** | OeSUT1-643 | **C** | OeLUS-277 | **T** |  |  |  |  |
| HRAISOUNI | OeACP2-257 | **Y** | OeLUS-277 | **W** | OeLUS-122 | **T** |  |  |  |  |
| ISTARKABELICA | OeACP2-688F | **C/DEL** | OeACP1-503 | **T** | OeSUT1-176 | **T** |  |  |  |  |
| KONSERVOLIA | OeSUT1-590 | **R** | OeLUS-259 | **R** | OeLUS-277 | **T** |  |  |  |  |
| KORONEIKI | OeACP2-103 | **S** | OeACP1-245 | **M** | OeACP2-257 | **T** |  |  |  |  |
| LECCINO | OeACP1-537 | **T/DEL** | OeACP2-28 | **Y** | OeLUS-122 | **T** |  |  |  |  |
| LUCQUES | OeACP2-1167 | **R** | OeSUT1-643 | **T/DEL** | OeSUT1-321 | **-** |  |  |  |  |
| MANZANILLAJAEN | OeSUT1-590 | **R** | OeACP1-181 | **M** | OeACP1-245 | **M** |  |  |  |  |
| MERHAVIA | OeACP1-306-312 | **T** | OeSUT1-643 | **C** | OeACP2-108 | **T** |  |  |  |  |
| MESKI | OeACP2-688A | **C/DEL** | OeSUT1-643 | **C** | OeACP1-181 | **C** |  |  |  |  |
| MORAIOLO | OeACP2-103 | **S** | OeACP1-245 | **A** | OeACP2-239B | **T/DEL** |  |  |  |  |
| NOSTRALERIGALI | OeSUT1-643 | **Y** | OeACP2-148 | **S** | OeACP1-306-312 | **-** |  |  |  |  |
| NOSTRANABRISIGHELLA | OeSUT1-566 | **C** | OeACP1-306-312 | **-** | OeSUT1-243 | **Y** |  |  |  |  |
| OLIVIERE | OeSUT1-176 | **G** | OeLUS-442 | **G** | OeACP1-537 | **T/DEL** |  |  |  |  |
| ORBETANA | OeACP2-103 | **S** | OeACP2-239B | **-** | OeACP2-257 | **T** |  |  |  |  |
| OTTOBRATICA | OeSUT1-243 | **Y** | OeLUS-442 | **S** | OeACP2-348 | **R** |  |  |  |  |
| PICHOLINEMAROCAINE | OeACP2-688A | **C/DEL** | OeSUT1-643 | **Y** | OeACP2-598C | **A/DEL** |  |  |  |  |
| PICUDO | OeSUT1-176 | **G** | OeACP1-537 | **T/DEL** | OeLUS-442 | **S** |  |  |  |  |
| RAIA | OeACP2-851 | **T** | OeSUT1-643 | **C/DEL** | OeACP1-306-312 | **-** |  |  |  |  |
| ROYALCAZORLA | OeSUT1-176 | **G** | OeACP2-1167 | **R** | OeLUS-442 | **S** |  |  |  |  |
| SEMIDANA | OeACP2-1167 | **R** | OeLUS-277 | **K** | OeSUT1-321 | **-** |  |  |  |  |
| SINOPOLESE | OeACP2-851 | **T** | OeACP2-348 | **G** | OeACP1-306-312 | **T/DEL** |  |  |  |  |
| VILLALONGA | OeSUT1-176 | **G** | OeACP1-537 | **T/DEL** | OeLUS-442 | **S** |  |  |  |  |
| ZAITUNA | OeACP2-688A | **C/DEL** | OeACP1-245 | **C** | OeLUS-442 | **G** |  |  |  |  |
| AMIGDALOLIA | OeACP1-407 | **Y** | OeSUT1-643 | **C** | OeACP2-1167 | **R** | OeLUS-277 | **T** |  |  |
| BLANQUETA | OeACP2-1167 | **R** | OeSUT1-643 | **C/DEL** | OeLUS-442 | **G** | OeACP1-245 | **A** |  |  |
| BORGIONA | OeACP1-407 | **Y** | OeSUT1-643 | **C** | OeACP2-1167 | **R** | OeLUS-277 | **T** |  |  |
| BOUTELLAIN | OeACP1-407 | **Y** | OeACP2-348 | **R** | OeACP1-245 | **M** | OeACP2-688A | **T** |  |  |
| CAROLEA | OeSUT1-643 | **T** | OeACP1-245 | **M** | OeACP2-348 | **R** | OeACP2-1120B | **-** |  |  |
| ITRANA | OeACP2-239B | **T/DEL** | OeSUT1-643 | **T** | OeACP1-245 | **A** | OeACP2-28 | **T** |  |  |
| IZMIRSOFRALIK | OeSUT1-259 | **T** | OeACP2-1167 | **G** | OeACP1-181 | **C** | OeACP1-407 | **T** |  |  |
| MANZANILLASEVILLA | OeACP2-688A | **C/DEL** | OeSUT1-643 | **T/DEL** | OeSUT1-176 | **K** | OeLUS-259 | **A** |  |  |
| PASSULUNARA | OeACP1-407 | **Y** | OeSUT1-643 | **C** | OeACP2-1167 | **R** | OeLUS-277 | **T** |  |  |
| PICHOLINE | OeACP2-598A | **A** | OeACP2-28 | **T** | OeACP1-503 | **K** | OeLUS-277 | **T** |  |  |
| PIANTONEMOGLIANO | OeACP1-407 | **Y** | OeSUT1-643 | **T** | OeSUT1-566 | **Y** | OeLUS-347 | **T** |  |  |
| CORNICABRA | OeACP2-1167 | **R** | OeSUT1-643 | **C/DEL** | OeACP2-598A | **-** | OeACP2-688A | **T** | OeACP2-688B | **T/DEL** |
| VERDALE | OeACP2-1167 | **R** | OeLUS-259 | **R** | OeSUT1-176 | **T** | OeACP2-372 | **T** | OeACP1-245 | **A** |
| ZALMATI | OeACP2-239B | **T/DEL** | OeACP1-537 | **T/DEL** | OeLUS-277 | **K** | OeACP1-407 | **T** | OeACP2-103 | **G** |

* Letters correspond to IUPAC code; DEL = deletion.
