# Supplementary material for: High Levels of Variation Within Gene Sequences of Olea europaea L
Source: Front Plant Sci. 2019 Jan 8;9:1932. doi: 10.3389/fpls.2018.01932 (PMC6331486; doi:10.3389/fpls.2018.01932)
Supplement: Table S7 — Summary of general linear model (GLM) and mixed linear model (MLM) results. For each phenotype, p-value, type of polymorphism, polymorphism position, and genotype occurrence are reported. [file Table_7.DOCX]

**Supplementary Table S7 |** Summary of General Linear Model (GLM) and Mixed Linear Model (MLM) results. For each phenotype, *p* value, type of polymorphism, polymorphism position and genotype occurrence are reported.

| **Phenotype** | ***p* values**  **GLM-MLM** | **Polymorphism** | **Polymorphism position (bp)** | **Genotype occurrence** |
| --- | --- | --- | --- | --- |
| FFW | 0.033-0.622 | ***OeACP1***  SNP C/T | 407 | 15 |
| FFW | 0.047-0.162 | ***OeACP2***  SNP T/A | 869 | 9 |

FFW: fresh fruit weight
